# Short- and long-term impact of hyperoxia on the blood and retinal cells’ transcriptome in a mouse model of oxygen-induced retinopathy

**DOI:** 10.1038/s41390-019-0598-y

**Published:** 2019-10-02

**Authors:** Magdalena Zasada, Anna Madetko-Talowska, Cecilie Revhaug, Anne Gro W. Rognlien, Lars O. Baumbusch, Teofila Książek, Katarzyna Szewczyk, Agnieszka Grabowska, Miroslaw Bik-Multanowski, Jacek Józef Pietrzyk, Przemko Kwinta, Ola Didrik Saugstad

**Affiliations:** 1 Department of Paediatrics, Jagiellonian University Medical College, Krakow, Poland; 2 Department of Medical Genetics, Jagiellonian University Medical College, Krakow, Poland; 3 Department of Paediatric Research, Oslo University Hospital Rikshospitalet, Oslo, Norway; 4 University of Oslo, Oslo, Norway

## Abstract

**Background:**

We aimed to identify global blood and retinal gene expression patterns in murine oxygen-induced retinopathy (OIR), a common model of retinopathy of prematurity, which may allow better understanding of the pathogenesis of this severe ocular prematurity complication and identification of potential blood biomarkers.

**Methods:**

A total of 120 C57BL/6J mice were randomly divided into an OIR group, in which 7-day-old pups were maintained in 75% oxygen for 5 days, or a control group. RNA was extracted from the whole-blood mononuclear cells and retinal cells on days 12, 17, and 28. Gene expression in the RNA samples was evaluated with mouse gene expression microarrays.

**Results:**

There were 38, 1370 and 111 genes, the expression of which differed between the OIR and control retinas on days 12, 17, and 28, respectively. Gene expression in the blood mononuclear cells was significantly altered only on day 17. *Deptor* and *Nol4* genes showed reduced expression both in the blood and retinal cells on day 17.

**Conclusion:**

There are sustained marked changes in the global pattern of gene expression in the OIR mice retinas. An altered expression of *Deptor* and *Nol4* genes in the blood mononuclear cells requires further investigation as they may indicate retinal neovascularization.

## Introduction

Retinopathy of prematurity (ROP), a proliferative retinal vascular disorder affecting premature infants, is currently a major cause of potentially preventable blindness in children.^1^ It is estimated that ROP affects 33.2% of neonates with birth weight < 1500 g.^2^ ROP develops in two phases; phase 1 is initiated by hyperoxia and exacerbated by lack of the essential factors provided in utero, followed by a destruction of existing vessels and arrest of normal vessel development. Phase 2 is driven by increased metabolic activity of the hypoxic retina, which leads to both revascularization and pathological neovascularization.^3^ An excessive abnormal compensatory vessel growth may result in fibrous scar development, and ultimately leads to retinal detachment.^4^ We believe that better understanding of the pathomechanisms behind ROP coupled with an early and effective method of the risk stratification of ROP development would allow to take true preventive actions and/or targeted treatment. Genome-wide microarray analysis provides a simultaneous examination of the expression of thousands of genes in a single experiment to create a comprehensive picture of dysregulated genes and pathways. A possibility to use the animal model that mimics key features of the disease provides invaluable tool for studying ROP underlying mechanisms not only in the blood, but also in the target tissue such as retina. The most widely used model is a murine model of oxygen-induced retinopathy (OIR).^5^ In mice, the retinas are mostly avascular after birth, and retinal vascularization occurs in the early neonatal period, resembling situation observed in the prematurely born infants. 5-day long continuous exposure to high oxygen levels imitates the biphasic retinal vascular response observed in the young infants with ROP.^6^ Up till now, exploration of the retinal gene expression pattern in the murine OIR model was assessed partially. Sato et al.^7^ evaluated only a selected set of genes related to angiogenesis and inflammation. Subsequent studies investigated expression of the entire genome, but at the selected time points—Ishikawa et al.^8^ and Yang et al.^9^ determined a profile of gene expression in murine retinas soon after end of hyperoxia, whereas Recchia et al.^10^ additionally verified gene expression in rodent retinas during neovascularization phase. The goal of this study was to identify potential ROP blood biomarkers and provide a more molecular-based understanding of ROP by comparing the gene expression profile at three time points (vaso-obliteration (P12), maximal neovascularization (P17) and during normalization of the retinal damage (P28)) in the blood mononuclear cells and retinas obtained from the mice with and without OIR. We hypothesized that supplemental oxygen as compared with air induced gene expression changes in the blood and retinal cells, which varied depending on the length of time following exposure to hyperoxia.

## Methods

### Murine model of OIR

The Norwegian Animal Research Authority approved all experiment protocols. The animals were cared for and handled in accordance with the Norwegian Legislation and Directive. OIR was induced in C57BL/6 mice (received from: Jackson Laboratories, Bar Harbor, ME) according to the protocol described by Smith et al.^6^ Briefly, mice were housed and bred at 24 °C at a 12:12 light/dark cycle with access to a standard rodent pellets and water ad libitum. On the postnatal day 7 (P7), all pups within each litter were earmarked with use of surgical micro-scissors. When a pup was picked up from the cage, it received a number drawn by an assistant from blinded tickets containing numbers from 1 to 8. The procedure was repeated until every pup had its own number within the litter. The smallest pups (according to their body weight on P7) out of litters of >8, or any abnormal pups (with visible morphologic abnormalities) were excluded from the experiment. Each litter with a nursing mother was then randomized either to hyperoxia (75% oxygen) or normoxia (21% oxygen) conditions (also by drawing a ticket either with a symbol N for normoxia or H for hyperoxia), then transferred in their cage into A-Chambers (BioSpherix Ltd, Parish, NY), where they were kept until day 12 (P12). Seventy-five percent oxygen concentration was achieved with a conventional oxygen mixer (oxygen (AGA AS, Oslo, Norway) plus room air). Each chamber was continuously monitored for oxygen concentration (O2-monitor, ProOX110 BioSpherix Ltd, Parish, NY), temperature and humidity (Thermometer/Hygrometer Clas Ohlson, Insjon, Sweden), and CO_2_ level (ProCO_2_ P120, BioSpherix Ltd, Parish, NY). An open water source kept air humidity at 40–50%. Every day there was a 10–20 min break in oxygen supply for airing the chamber and checking the mice. Nursing dams stayed with their own litters throughout the entire experiment. The cages containing pups with their mothers were removed from the hyperoxia/normoxia chambers on day P12. Before removing cages with mice from A-Chambers, one more randomization was performed; namely tickets with symbols, indicating the predicted harvesting day and type of analyses were prepared, then they were randomly assigned to each pup from either hyperoxia or normoxia group. 1/3 of the pups were immediately anesthetized (zolazepam/tiletamine/xylazine/fentanyl standard ZRF cocktail, 0.1 ml/10 g intraperitoneally), their gender was assessed, and the blood samples obtained by cardiac puncture were collected to 1 ml syringes (Terumo U40/1 ml, needle 0.33 x 12), flushed with EDTA, then processed within 1 h, as described below. Then mice were killed by aorta cut and their eyes were enucleated. The rest of the litters with nursing mothers returned to room air and stayed under normal conditions till P17, or P28, according to their randomly selected groups. Their blood and retinas were collected and processed the same way as described above.

### Murine blood mononuclear cells purification and total RNA isolation

Blood sample was diluted with equal amount of sterile phosphate-buffered saline (PBS) solution (GE Healthcare Life Sciences). One milliliter of Ficoll-Paque (GE Healthcare Life Sciences) was inserted into a 2 ml Eppendorf centrifuge tube. Diluted blood sample was layered carefully onto the Ficoll-Paque. Then sample was centrifuged at 400 gav for 30 min at 18 °C. After removing an upper layer, a lymphocyte layer was transferred into a new 2 ml Eppendorf tube, filled with PBS solution, mixed and afresh centrifuged at 100 gav for 10 min at 18 °C. Then the supernatant was discarded carefully not to disrupt the pellet of the peripheral blood mononuclear cells (PBMC). Subsequently, PBMCs were washed again with PBS solution (centrifugation repeated under the same conditions). Afterwards 800 µl of lysis solution and 50 µl of sodium acetate (Mouse RiboPure™-Blood RNA Isolation Kit; Invitrogen; Thermo Fisher Scientific) was added, the cells were suspended by manual pipetting, then the samples were placed immediately at −80 °C. Total RNA was isolated with the use of Mouse RiboPure™-Blood RNA Isolation Kit (Invitrogen; Thermo Fisher Scientific) according to the manufacturer’s instructions. RNA concentration was measured with the use of NanoDrop Spectrophotometer (NanoDrop ND-1000; Thermoscientific, Waltham), and RNA quality was determined by TapeStation 4200 (Agilent, Santa Clara).

### Obtaining retinas and total RNA isolation

Whole retinas free of additional tissues were isolated immediately under a dissecting microscope, and put into vials with RNA*later*® Stabilization Solution (Thermo Fisher Scientific), kept for 24 h in + 4 °C and stored in a −80 °C freezer until further use. To isolate sufficient total RNA for microarray analysis, two retinas from the same mouse were pooled. Retina tissues were then disrupted with the use of TissueRuptor handheld rotor-stator homogenizer (Qiagen, Hilden, Germany) and total RNA from the retina cells was processed by RNeasy Micro Kit (Qiagen, Hilden, Germany) as described by the manufacturer. RNA concentration was measured with the use of NanoDrop Spectrophotometer (NanoDrop ND-1000; Thermoscientific, Waltham), and RNA quality was determined by TapeStation 4200 (Agilent, Santa Clara).

### Validation of the model

Eyes from randomly selected 36 pups both from normoxia and hyperoxia groups, 1/3 from each harvesting time point, were used for the verification of the model, according to the methods described in details elsewhere.^3^ Briefly, the eye was enucleated and fixed with 4% Paraformaldehyde Solution (Boster Biological Technology, Pleasanton CA). Then under the dissecting microscope the retina was isolated, incubated in 0.5% Triton X-100 (Sigma, St. Louis, MO) and stained in the lectin solution with Alexa Fluor 594–I21413, Molecular Probes. Then under a dissecting microscope retinas were mounted on a microscope slide and cover slipped with SlowFade medium (Thermo Fisher Scientific, Waltham, MA). Images were obtained using the Zeiss AxioCam MRc, Zeiss AxioObserver.Z1 microscope and AxioVision 4.8 software. Using Adobe Photoshop CS6, the percentage of avascular retina, as well as neovascularization fraction was calculated by an independent researcher.

### Microarray analyses

One-hundred nanograms of total RNA was used for each single experiment with use of SurePrint G3 Mouse Gene Expression 8x60K Microarrays (Agilent, Santa Clara) to study the mRNA expression. Microarray gene expression experiment was performed according to the manufacturer’s protocol (Two-Color Microarray-Based Gene Expression Analysis—Low Input Quick Amp Labeling ver.6.5). The universal mouse reference RNA (Agilent Technologies, CA) was used as an internal control. After experiment, the microarrays were scanned by SureScan Microarray Scanner (Agilent, Santa Clara) and data was extracted using Feature Extraction Software (Agilent, Santa Clara). Details of the microarray experiment are available on-line (https://www.ncbi.nlm.nih.gov/geo/query/acc.cgi?acc=GSE130400, https://www.ncbi.nlm.nih.gov/geo/query/acc.cgi?acc=GSE130347)

### Quantitative real-time PCR (qRT-PCR) verification of gene expression

To verify the microarray gene expression data, ten genes were randomly selected for a method validation using qRT-PCR. The genes represented well described and characterized murine genes chosen from the popular databases, such as Ensembl, NCBI, or DAVID. The genes specific for retinas included*: Cidea, Etnppi, Spink13, Gabrb1, Bfsf2, Tnnt2, Adm, Gfap, Nupr, Actg2*. The primers and probes were commercially available (Thermo Fischer Scientific, USA). The complementary DNA was synthesized using SuperScript III kit (Thermo Fischer Scientific) according to the manufacturer’s protocol. The qRT-PCR analysis was performed using Applied Biosystems 7500 Real-Time PCR System and TaqMan Universal Master Mix II, no UNG (Thermo Fischer Scientific). The qRT-PCR reactions were performed in 96-well plates at 95 °C for 10 min, followed by 40 cycles of 95 °C for 15 s and 62 °C for 1 min. All samples were run in duplicates. Gene expression was calculated by ΔΔCt method, normalized against the endogenous control gene *Gapdh* and for each gene fold change (FC) values were calculated as previously described.^11^ In blood samples, the same validation method as described above was performed in seven randomly selected genes, due to very limited amount of blood and RNA obtained from the small pups.

### Statistical analysis

Gender distribution was evaluated with chi-square test. A probability value of < 0.05 was considered statistically significant. JMP® 13.1.0 (SAS Institute Inc., 2016) was used for statistical analysis.

Microarray data were analyzed by GeneSpring GX software (Agilent, Santa Clara). Raw microarray data were normalized, then moderated *t*-test was applied to compare gene expression between the study groups. The results were corrected for multiple testing by the Benjamini–Hochberg procedure. A 0.05 significance level of the adjusted *p*-values was used. The genes, the expression of which was significantly altered during the experiment, were further investigated using established databases, such as Gene Cards (http://www.genecards.org), Mouse genome informatics (http://www.informatics.jax.org), and NCBI gene (http://www.ncbi.nlm.nih.gov), Ensembl (http://www.ensembl.org/Mus_musculus).

## Results

### Animal model of OIR

**Fig. 1:**
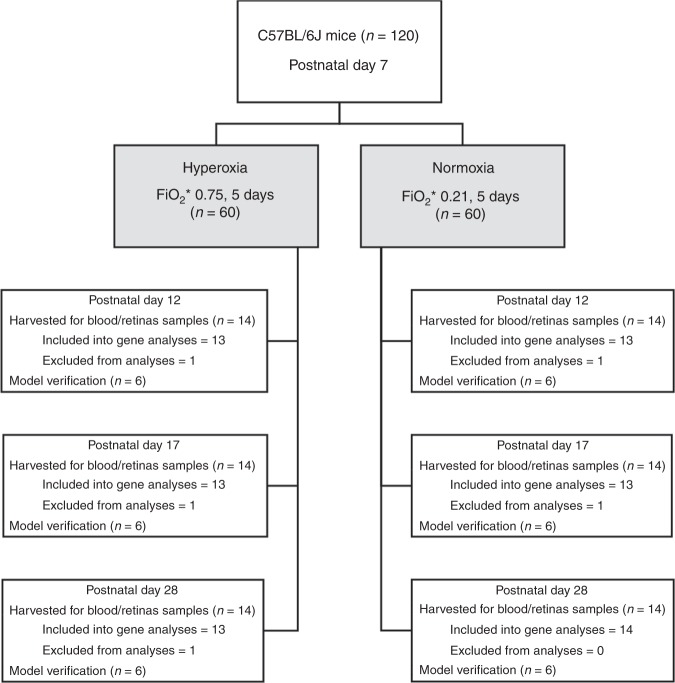
Flowchart of the study. Experimental design and experimental groups. *FiO_2_—fraction of inspired oxygen

On P7, 120 C57BL/6J mice underwent the experiment—60 were randomly assigned to the OIR group, and the other 60 formed the control group. All animals survived the experiment, and were further randomized according to the day of tissue harvesting (P12, P17, or P28). Six mice from each group from every experimental time point were used for model verification. Out of 84 animals assigned to the gene expression analysis, five were excluded due to insufficient RNA concentration and low sample quality (Fig. 1).

**Fig. 2:**
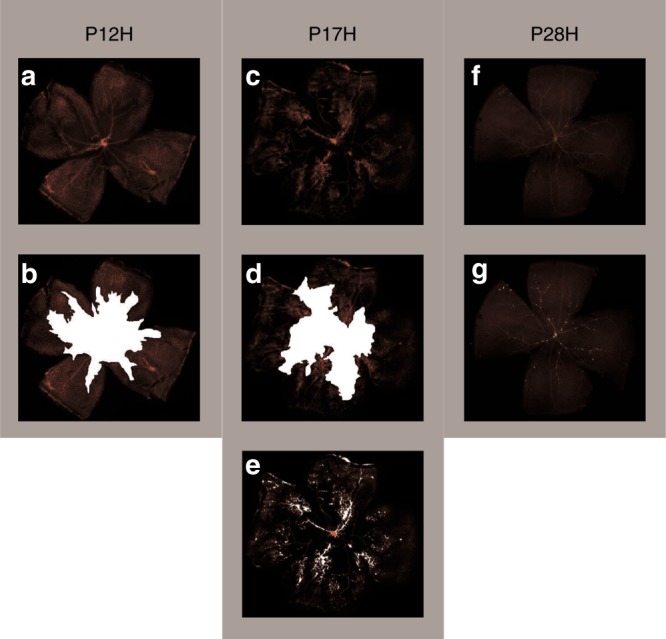
Retinal flat mounts from OIR groups at different time points: P12H (postnatal day 12, right after hyperoxia), P17H (postnatal day 17, 5 days after hyperoxia), P28H (postnatal day 28, 16 days after hyperoxia). The retinal vessels were stained with lectin. P12H: **a** vaso-obliteration showed as a large avascular zone in the central retina, **b** the avascular area presented in white; P17H: **c** maximal pathological retinal neovascularization (NV). The retinal vessels were dilated and there were tufts of NV on the surface of the retina, **d** the avascular area shown in white, **e** the areas of neovascularization shown in white; P28H: **f** neovascular changes regression, **g** the remained areas of neovascularization are shown in white

The two groups did not differ in gender distribution (Table 1). There were no significant weight differences between the groups when entering experiment (P7); however, OIR group revealed transient significant extrauterine growth retardation on P12, which normalized after day P17 (Table 2). Retinal vascular staining confirmed oxygen-related damage of the retinas in the OIR group (Fig. 2).

**Table 1:** Characteristics of dams and litters in the study groups

	Hyperoxia			Normoxia			Pearson chi-square *p*—for gender distribution
	No of dams/litters (*n*)	Male pups (*n*)	Female pups (*n*)	No of dams/litters (*n*)	Male pups (*n*)	Female pups (*n*)	
In total	18	20	19	16	21	19	0.5400
P12	12	5	8	12	8	5	0.2393
P17	10	10	3	10	7	6	0.2162
P28	10	5	8	9	6	8	0.8163

**Table 2:** Mean weight in selected time points in OIR and control animals, whose tissues were further proceeded for the transcriptome analysis

	OIR group	Normoxia group	*p* (two sided *t*-test)
Weight on postnatal day (P) [g]:			
P7 (mean, (SD))	4.13 (0.41)	3.98 (0.59)	0.2092
P10 (mean, (SD))	5.31 (0.54)	5.29 (0.53)	0.8675
P12 (mean, (SD))	5.58 (0.54)	6.06 (0.56)	0.0003
P14 (mean, (SD))	6.13 (0.68)	6.92 (0.80)	0.0004
P17 (mean, (SD))	7.03 (0.78)	7.53 (0.84)	0.0268
P21 (mean, (SD))	8.49 (1.19)	8.86 (1.02)	0.3939
P28 (mean, (SD))	14.96 (1.93)	16.07 (1.34)	0.1286

### Overall gene expression changes in retina

**Fig. 3:**
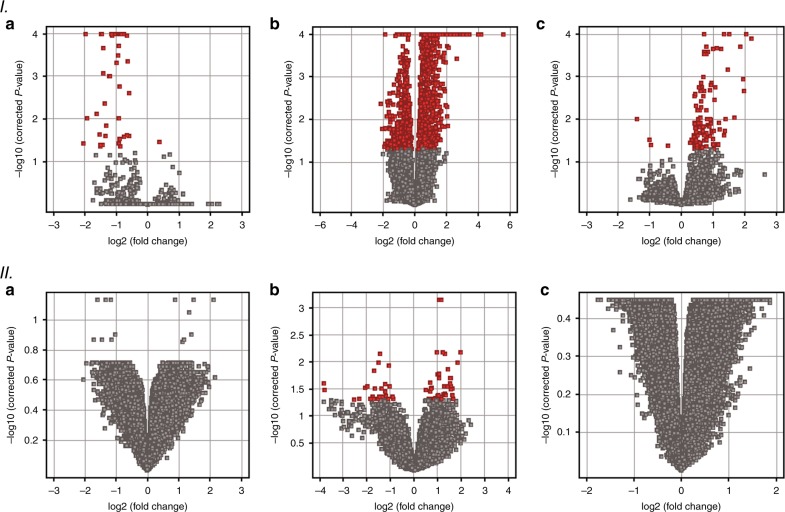
Volcano plots demonstrating that OIR was associated with alterations of specific genes in all analyzed time points (**a**—P12, **b** —P17, **c**—P28). I—gene expression changes in retina, II—gene expression changes in blood. The vertical axis value—negative log of the *p*-values. The horizontal axis—log2 change (FC—fold change) between the two experimental conditions (OIR vs. normoxia). The red dots represent genes, whose expression was significantly changed between the study vs. control groups (corrected *p*-value < 0.05)

Microarray analysis revealed significant differences between OIR and control group in gene expression in murine retinas in all experimental time points (Table 3 and Fig. 3—part 1). Overall, there were 39 genes (38 genes with annotated gene symbol) differentially expressed on day P12, 1478 genes (1370 genes with annotated gene symbol) on P17, and 114 genes (111 genes with annotated gene symbol) on P28. On P12, the predominant fraction of the significantly altered genes showed reduced expression, whereas the majority of the genes demonstrated increased expression in later days, both P17 and P28, in the OIR group when compared with the controls. We observed a shift in gene expression pattern in OIR murine retinas, starting from a significantly diminished gene expression immediately after hyperoxia exposure through a peak gene overexpression on P17, when maximal neovascularization was observed, till gradual decline of gene overexpression on P28 (Fig. 3).

**Table 3:** Summary of the number of differentially expressed genes in retina tissue and blood between OIR and control group at different time points (cutoff: moderated *t*-test corrected *p*-value < 0.05)

No of differentially expressed genes with annotated gene symbol (no of differentially expressed microarray probes)	P12		P17		P28	
	Retina	Blood	Retina	Blood	Retina	Blood
All	38 (39)	0 (0)	1370 (1478)	49 (63)	111 (114)	0 (0)
Overexpressed genes	1	0	855	34 (38)	107	0
Underexpressed genes	37	0	515	15 (25)	4	0

The genes with the most diverse expression defined by the highest fold change (FC) values are listed in Tables 4 and 5.

**Table 4:** Top list of downregulated genes in retina and blood based on the fold change in their expression between OIR and control mice at different time points

Time point	Gene symbol	Log2FC	Corrected *p*-value	Gene name
Retina				
P12	*Ccm2l*	−4.15	0.0374	Cerebral cavernous malformations 2 protein-like
	*Abcc9*	−3.93	2.0026E-6	ATP-binding cassette subfamily C member 9
	*Adcy4*	−3.10	0.0095	Adenylate cyclase type 4
	*Arap3*	−2.90	0.0075	Arf-GAP with Rho-GAP domain, ANK repeat and PH domain-containing protein 3
	*Mmrn2*	−2.85	0.0230	Multimerin-2
	*Robo4*	−2.82	0.0433	Roundabout homolog 4
	*Cd34*	−2.81	0.0400	Hematopoietic progenitor cell antigen CD34
	*Adgre5*	−2.76	2.6282E-9	Adhesion G-protein-coupled receptor E5
	*Ptprb*	−2.69	2.0025E-6	Receptor-type tyrosine-protein phosphatase beta
	*Ets1*	−2.66	0.0400	Protein C-ets-1
P17	*Cidea*	−4.44	0.0043	Cell death activator CIDE-A
	*Kcne1l*	−4.17	0.0169	Potassium voltage-gated channel subfamily E regulatory beta subunit 5
	*Kcnd2*	−3.82	0.0430	Potassium voltage-gated channel subfamily D member 2
	*Spink13*	−3.78	0.0166	Serine protease inhibitor Kazal-type 13
	*Etnppl*	−3.69	0.0371	Ethanolamine-phosphate phospho-lyase
	*Spc25*	−3.64	7.0628E-9	Kinetochore protein Spc25
	*Gabrb1*	−3.63	0.0163	Gamma-aminobutyric acid receptor subunit beta-1
	*Tacr3*	−3.33	0.0477	Neuromedin-K receptor
	*Scn11a*	−3.31	0.0350	Sodium channel protein type 11 subunit alpha
	*Gpr101*	−3.30	0.0039	Probable G-protein-coupled receptor 101
P28	*Fam219aos*	−2.62	0.0099	Family with sequence similarity 219, member A, opposite strand
	*Grip1*	−1.95	0.0405	Glutamate receptor-interacting protein 1
	*Crabp1*	−1.33	0.0417	Cellular retinoic acid-binding protein 1
Blood				
P17	*Gm1043*	−5.02	0.0481	Predicted gene 1043
	*Gm29260*	−4.21	0.0298	Predicted gene *29260*
	*Tbc1d17*	−3.63	0.0481	TBC1 domain family member 17
	*Nol4*	−3.23	0.0146	Nucleolar protein 4
	*Gm4013*	−3.13	0.0304	Predicted gene *4013*
	*Gm3970*	−2.82	0.0102	Predicted gene *3970*
	*Ccl20*	−2.76	0.0434	C-C motif chemokine 20
	*Cers4*	−2.34	0.0253	Ceramide synthase 4
	*Nbeal1*	−2.08	0.0115	Neurobeachin-like 1
	*Serpina11*	−2.03	0.0282	Serpin A11

**Table 5:** Top list of upregulated genes in retina and blood based on their expression fold change between OIR and control mice at different time points

Time point	Gene symbol	Log2FC	Corrected *p-*value	Gene name
Retina				
P12	*Mri1*	1.29	0.0338	Methylthioribose-1-phosphate isomerase
P17	*Esm1*	49.02	1.3345E-11	Endothelial cell-specific molecule 1
	*Edn2*	18.60	1.8853E-5	Endothelin-2
	*Bcl3*	16.71	1.4607E-8	B-cell lymphoma 3 protein homolog
	*Apln*	16.05	3.7819E-9	Apelin
	*C4b*	11.10	1.3346E-11	Complement C4-B
	*Pcolce*	10.65	6.6323E-11	Procollagen C-endopeptidase enhancer 1
	*Cebpd*	10.47	1.9108E-8	CCAAT/enhancer-binding protein delta
	*Adm*	9.68	7.5001E-11	Adrenomedullin
	*Tnnt2*	8.46	3.2014E-8	Troponin T, cardiac muscle
	*Gfap*	8.06	7.0122E-6	Glial fibrillary acidic protein
P28	*Bcl3*	4.62	1.2942E-4	B-cell lymphoma 3 protein homolog
	*C4b*	4.17	2.9285E-7	Complement C4-B
	*Lad1*	3.92	0.0023	Ladinin-1
	*Apln*	3.92	0.0012	Apelin
	*Pcolce*	3.64	2.0317E-4	Procollagen C-endopeptidase enhancer 1
	*Fgf2os*	3.22	0.0091	Uncharacterized protein
	*Tnnt2*	2.86	2.8065E-5	Troponin T, cardiac muscle
	*Cebpd*	2.77	6.8232E-4	CCAAT/enhancer-binding protein delta
	*Lyz1*	2.64	0.0099	Lysozyme C-1
	*Glycam1*	2.61	0.0187	Glycosylation-dependent cell adhesion molecule 1
Blood				
P17	*Taf4*	3.11	0.0403	Transcription initiation factor TFIID
	*Sec22a*	2.96	0.0195	Vesicle-trafficking protein SEC22a
	*Trmt1l*	2.91	0.0195	tRNA methyltransferase 1 -like protein
	*ND2*	2.50	0.0070	NADH-ubiquinone oxidoreductase chain 2
	*Rrp1b*	2.32	0.0067	Ribosomal RNA processing protein 1 homolog B
	*Trp53*	2.25	6.9234E-4	Cellular tumor antigen p53
	*Fam20b*	2.23	0.0481	Glycosaminoglycan xylosylkinase
	*Hmg20b*	1.98	0.0067	SWI/SNF-related matrix-associated actin-dependent regulator of chromatin subfamily E member 1-related
	*Gbf1*	1.66	0.0304	Golgi-specific brefeldin A-resistance factor-1
	*Mkln1*	1.60	0.0354	Muskelin

### Overall gene expression changes in blood

There were significant differences in gene expression only on P17 in OIR mice in comparison to the control animals in the PBMC analysis (Fig. 3). Out of 63 genes (49 genes with known official Gene symbols), around two-thirds were significantly upregulated, whereas one-third was downregulated (Table 3 and Fig. 3—part 2). Two genes, *Deptor* and *Nol4* showed decreased expression both in the blood and retinal tissues on day P17 (FC_retina_ < −3; FC_blood _< −2).

### Quantitative real-time PCR

**Fig. 4:**
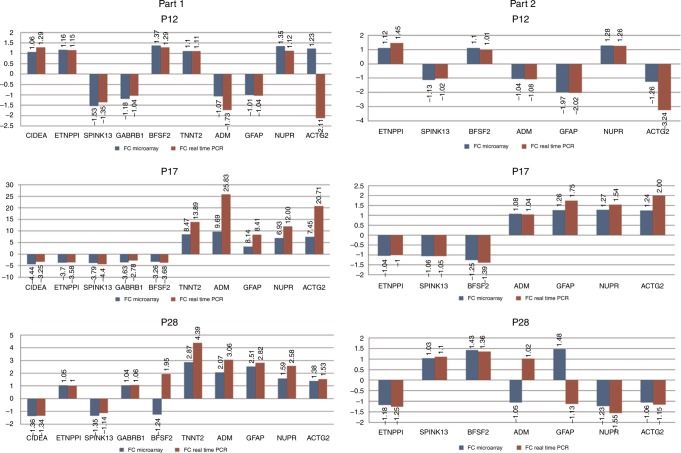
Validation of microarray study by real-time PCR technique in all analyzed time points. Part 1—gene expression in retina tissue, part 2—gene expression in blood

The validation procedure performed for genes randomly selected before experiments did not reveal significant differences between the results obtained with use of microarrays as compared with real-time PCR method (*t*-test *p-*values for each time point > 0.05). Detailed results for blood and retina tissue are shown in Fig. 4.

## Discussion

The goal of the study was to identify short- and long-term molecular alterations in the blood and retina after exposure to high levels of inhaled oxygen using a well-characterized murine model of oxygen-induced retinopathy. The nature of the study was mainly exploratory.

On P12, the genes with the most decreased expression were those associated with angiogenesis (*Ccm2l*, *Mmrn2*, *Ets1*, *Ptprb*, *CD34*, *Robo4*), cell shape rearrangement (*Arap3*), ion channel formation (*Abcc9*), as well as receptors involved in cell-to-cell interactions (*Adgre5*). Interestingly, some of the genes regulating angiogenesis were first underexpressed on P12, then they were overexpressed on P17 to finally normalize their expression on P28. An example of such gene response, the expression of which reversed between P12 and P17, was *Cd34* gene. *Cd34* encodes a sialomucin CD34, ubiquitously expressed in the luminal membrane of the endothelial cells, which promotes angiogenesis.^12^ CD34 transcript and protein levels are increased during human angiogenesis.^13^ According to Siemerink et al.^13^ its expression promotes pathological neovascularization in a murine OIR model by enhancing epi-retinal tuft formation.

On P17, the most profound decrease in expression was observed in the genes associated with: apoptosis (*Cidea*), metabolism (*Etnppl*), cell division (*Spc25*), ion channels (*Kcne1l, Kcnd2, Gabrb1, Scn11a*), and receptor components (*Garb1, Tacr3, Gpr101*), as well as serine protease inhibitors (*Spink13*).

On the same day, P17, the top overexpressed genes were *Esm1* and *Edn2* genes. *Esm1* encodes endothelial cell-specific molecule 1 (Esm1). Animal model showed that Esm1, in cooperation with vascular endothelial growth factor (VEGF), plays an important role in both physiologic and pathological angiogenesis and vascular permeability. It is also involved in some inflammatory processes by regulation of leukocytes extravasation.^14^

*Edn2* gene, which showed 18.6-fold increased expression on P17, encodes endothelin (ET) 2, a potent vasoconstrictor that contributes to regulation of the retinal blood flow, and may exert mitogenic, pro-oxidative, and proinflammatory effects.^15^ Our results of *Edn2* overexpression confirmed previously published findings of increased abundance of *Edn2* transcripts in various retinal injuries.^16^ A pharmacological blockade of the ET system prevented pathological neovascularization in a murine model of OIR.^17^ Other genes that showed increased expression in our study were also evaluated elsewhere. Inhibition of adrenomedullin, a peptide involved in vasodilation, angiogenesis and vascular leakage, encoded by *Adm* gene, highly overexpressed in our study, was identified as a potential target of new therapies in diabetic retinopathy.^18^ On the other hand, an increased expression of *Gfap* gene might indicate reactive gliosis as a reaction to retinal impairment.^19^

In previous studies, murine retinal neovascularization as visualized in the tissue samples spontaneously regressed between P17 and P25.^3^ However, our results indicated that despite almost complete disappearance of the visible neovascular changes, the alterations in gene expression in the retinal cells remained. In our study, we also examined gene expression in the retinas from mice on P28, which in mouse terms was considered a "young" adult or in human terms, at least an adolescent. We observed sustained overexpression of the several important genes (*Apln, Pcolce, Cebpd, Bcl3, Tnnt2, C4b*) from P17 to P28. *Apln* gene encodes a peptide apelin that stimulated angiogenesis and induces retinal neovascularization in the VEGF-independent manner.^20^ It was hypothesized, that apelin, after binding to its APJ receptor, promotes nitric oxide production through PI3K⁄Akt signaling and nitric oxide synthase activation.^21^ Apelin production increases in response to hypoxia and transcription enhancement by hypoxia-inducible factor-1.^22^ Of note, apelin was also found in vitreous humor of the patients with diabetic retinopathy.^23^ Interestingly, apelin knockout mice treated with VEGF showed impaired angiogenesis,^24^ therefore we believe that development of apelin/APJ inhibitors might represent a new therapeutic strategy to prevent pathological neovascularization in ROP. *Tnnt2* encodes a troponin T type 2, which might control excessive angiogenesis in hypoxic conditions.^25^ *Pcolce* gene encodes procollagen C-endopeptidase enhancer protein that is required for endothelial cell lumen formation.^26^ *Cebpd* gene encodes a transcription factor CCAAT/enhancer-binding protein D, associated with immune response^27^, as well as angiogenesis.^28^ *C4b* encodes complement C4-B involved in a variety of immune processes. B-cell leukemia/lymphoma 3 (*Bcl3*) gene serves as death promoter.^29^ We hypothesized that its increased expression on P17 corresponded to massive apoptosis of damaged retinal cells. Additionally, on P28, observed induction of genes involved in apoptosis might result in apoptosis of the endothelial cells, causing the already formed neo-vessels to regress. This observation was in accordance with the previously reported studies highlighting the role of apoptosis of the endothelial cells in pathological vascularization regression.^30^ Other genes highly overexpressed on P28 were these engaged in the inflammatory processes: *Glycam1* encodes a proteoglycan ligand for L-selectin, which in turn exerts an anti-inflammatory effect by prevention of monocyte extravasation,^31^ *Lyz1* encodes lysozyme, a potent antimicrobial protein. The basement membrane anchoring filament protein Lad1, encoded by *Lad1* gene, contributes to the epithelial stability.^32^ Lad1 gene expression is regulated by both lipopolysaccharide stimulation and glucocorticoid receptor activation.^33^ Overexpression of the genes associated with various aspects of immune response might indicate a local inflammatory process, which continue in the damaged retina over time. Moreover, we observed an increased expression of *Fgf2os* gene, encoding fibroblast growth factor 2 opposite strand, which was hypothesized to aid in preservation of the neuroretinal function during ischemic retinopathy.^34^

Microarray analysis of the blood mononuclear cells revealed alterations in gene expression pattern only on P17. Our results indicated that examination of the PBMC’s genome could not provide a good early biomarker of OIR, which further confirmed our findings in the previous human study on ROP.^11^ Lack of the altered gene expression on P28 indicated transient nature of the gene expression changes in the blood MNCs caused by hyperoxia. Moreover, alterations in gene expression present at retina tissue may appear in PBMCs with a shift of time, therefore they are not visible at the studied time points. It is also possible, that the applied experimental procedure that caused only subtle changes in gene expression at P12 and P28 in the target tissue (retina) was not potent enough to trigger changes visible in the blood cells. It should also be taken into consideration that when performing a statistical analysis with an amendment to multiple comparisons (in the presented experiments Bonferroni correction was applied), small differences in gene expression are overlooked. Finally, it is worth to mention that many of the observed gene expression changes may be tissue-specific. Consequently we may assume, that not all changes in expression present in the retina are reflected in PBMCs.

The genes with the most decreased expression in the PBMC included the ones encoding proteins involved in various cellular mechanisms (*Serpina11, Tbc1d17,Cers4*), immune response (*Ccl20*), as well as genes encoding long non-coding RNA (*Gm29260, Gm4013*).

There were two genes that showed significantly reduced expression with FC < −2.0 both in the retinal and blood cells, on P17. These were *Deptor* and *Nol1* genes. *Deptor* gene encodes DEP Domain-Containing mTOR-Interacting Protein, which is a negative regulator of mammalian target of rapamycin (mTOR) signaling pathways. The mTOR-signaling pathways were previously shown to be profoundly interrelated with several mechanisms crucial for retinopathy progression, including oxidative stress, inflammation, hypoxia, angiogenesis, and proliferation/fibrosis,^35,36^ Therefore, decreased *Deptor* gene expression in both blood mononuclear cells and retina might not only mark encountered oxidative stress, but also indicate effects of mTOR-signaling pathway overactivation, including neovascularization.

Nucleolar protein 4 (*Nol4*) gene might be involved in a process of determining cell specificity.^37^ As a tumor suppressor gene showed tissue-specific distribution,^37^ it was considered as a potential biomarker of head and neck cancer, as it was highly hypermethylated in the patients suffering from these malignancies,^38,39^ Its role in the OIR would need to be elucidated.

The genes overexpressed on P17 in the blood were different from these upregulated in the retinas at the same time point, and mostly they were involved in transcription and its regulation and double-strand DNA break repair (*Aff3, Baz1b,Dlx5, Hmg20b, Lipin2, Nelfe, Trp53, Zfp426*). There was no surprise to find overexpression of *Trp53* gene since that was an indicator of DNA damage caused by hyperoxia.^40^

### Limitations

Although our experiment included a sufficient number of animals and the gene expression analysis was validated and confirmed, we are aware of some study limitations. The model of continuous hyperoxia exposure for 5 days is more similar to the former cases of ROP associated with treatment of neonates with very high oxygen levels. Future studies with a model more closely mimicking the clinical situation in preterm infants, such as a rat model of oxygen-induced retinopathy, are required to confirm the obtained results. Moreover, we measured only the ambient oxygen concentration within the chambers. We did not check SpO2 or pO2 in the mice subjected to hyperoxia during the hyperoxic period (in order to more precisely monitor hyperoxic exposure). Additionally, we observed transient extrauterine growth retardation in our OIR mice, which might have affected their phenotype. However, we did not observe mice weighing <6 g on P17, that according to Connor et al.^3^ might have profoundly altered the vascular phenotype. Validation of gene expression changes was performed only for selected genes. We understand that we presented gene expression results for the entire retina, which consisted of a several types of cells. Our study provided a starting point for further genetic analysis of ROP, in which we should investigate regulatory mechanisms of gene expression such as DNA methylation and/or protein synthesis. In addition our analysis was based predominantly on the molecular studies, we believe that future experiments should include functional data, both in vitro and/or in vivo provided in the specific cell types or in whole retina.

## Conclusions

Our mRNA expression analysis showed that particular OIR stages in mice were associated with specific changes in the pattern of gene expression in the retinas and allowed for selection of the candidate genes that may be the subject of further, more targeted studies (i.e., proteomics). Moreover, it also revealed, that transcriptome blood analysis might not be an early biomarker of OIR development, although the altered expression of *Deptor* and *Nol4* gene would require further investigation as they might become blood indicators of retinal neovascularization. Our work provided new evidence of the critical role of excessive tissue oxygenation that resulted in gene expression changes during the course of each stage of retinopathy of prematurity. The altered gene expression in retinas might present a long-lasting effect of a prior hyperoxia, the subsequent consequences of which would have to be determined in the future studies.
